# An Optimized Terpene Profile for a New Medical Cannabis Oil

**DOI:** 10.3390/pharmaceutics14020298

**Published:** 2022-01-27

**Authors:** Valentina Maggini, Lorenzo Calvi, Tommaso Pelagatti, Eugenia Rosaria Gallo, Celine Civati, Carlo Privitera, Flavio Squillante, Paolo Maniglia, Domenico Di Candia, Roberto Spampatti, Fabio Firenzuoli

**Affiliations:** 1 Research and Innovation Center in Phytotherapy and Integrated Medicine (CERFIT), Careggi University Hospital, Via Delle Oblate 4, 50141 Florence, Italy; lorenzocalvi@yahoo.it (L.C.); tommaso@farmaciatili.it (T.P.); eugenia.gallo@unifi.it (E.R.G.); 2 Farmacia Tili, Piazza Vittorio Veneto 32, 22036 Erba, Italy; civati.celine@gmail.com (C.C.); squillante.flavio@tiscali.it (F.S.); roberto@farmaciatili.it (R.S.); 3 Progetto MediCOmm s.r.l.s., C.da due Fontane s.n., 93100 Caltanissetta, Italy; carloprivitera@progettomedicomm.com; 4 Department of Anesthesia and Intensive Care Medicine, Alessandro Manzoni Hospital, Azienda Socio-Sanitaria Territoriale Lecco, 23900 Lecco, Italy; maniglia@iol.it; 5 Department of Biomedical Sciences for Health, Section of Legal Medicine, University of Milan, 20133 Milan, Italy; domenico.dicandia@unimi.it

**Keywords:** *Cannabis sativa* L., medical use, oil preparation, extraction method, cannabinoids, monoterpenes, sesquiterpenes, oxidized terpenes

## Abstract

The purpose of this analytical study was to develop an advanced formulation of medical Cannabis oil (MCO) comparing the chemical profile of different extracts obtained with two existing methods (SIFAP and CALVI) and one original upgraded (CERFIT) method. Preparation methods were applied with varying solvent, temperature, and duration of the decarboxylation and extraction steps. HPLC-MS/MS TSQ and GC/FID-HS analyses were performed to investigate cannabinoid and terpene contents in the three oil extracts. Cannabinoids profile remained comparable between the formulations. CERFIT extracts exhibited a superior quantity of total terpene hydrocarbon forms (e.g., limonene and α-pinene) with no degradation occurrence (i.e., oxidized terpenes not quantifiable). Thus, this new method optimized the phytochemical profile of the MCO presenting a value opportunity to obtain a standardized high-level therapeutic product.

## 1. Introduction

Cannabis, dating back thousands of years, has been utilized in treating various conditions and showed a pleiotropic effect [1]. Nowadays, the therapeutic potential of medical Cannabis (MC) is recognized, and patients have started to consider it as a worthwhile alternative [2]. From Canada to Australia, more than thirty countries (including most U.S. states) have invested in medical cannabis programs and over two million users are registered [3].

Cannabis is prescribed for specific medical conditions (often “intolerant” or “not-responder” to first line treatments), including chronic and cancer-related pain, nausea and vomiting produced by chemotherapy, HIV-related cachexia, multiple sclerosis spasticity, Tourette’s syndrome, glaucoma and pediatric epilepsy as well as there are emerging promises for the treatment of insomnia, depression, anxiety, autism and Alzheimer disease [4,5,6].

On the other hand, the physicians are reticent to prescribe MC not due to the need for training but also for the lack of standardized formulations in terms of bioactive compounds, in particular for the galenic preparations that should conversely permit one to take advantage of the therapeutic potential of MC phytocomplex [7]. The most studied MC molecules (Table 1) include Δ9-tetra-hydrocannabinoic acid (Δ9-THCA) (that is decarboxylated to Δ9-THC by heat and can further degrade to cannabinol), cannabidiolic acid (CBDA) and its decarboxylated metabolite CBD [8,9,10]. Cannabinol (CBN) is usually quantifiable and considered a product of THC oxidation [11]. Variability in cannabinoid concentrations has been reported in magistral preparations of MC extracted with different protocols [12]. Moreover, the phytocannabinoids are reported to interact with two other important compound classes, terpenes and flavonoids [13,14], thus determining the so-called entourage (synergistic) effect [14,15,16]. In the Cannabis plant, volatile monoterpenes, diterpenes, triterpenes (10, 20 and 30 carbon atoms, respectively) are present, as are sesquiterpenes (15C) that could contribute to Cannabis anti-inflammatory and antinociceptive effects [15,17]. Limonene, β-myrcene, and α-pinene represent the major Cannabis monoterpene components and β-caryophyllene is one of the predominant sesquiterpenes. A-pinene inhibits the activity of acetylcholinesterase in the brain, thus minimizing the cognitive dysfunction caused by THC intoxication [18]. Limonene is reported to boost serotonin and dopamine levels, thereby miming the anxiolytic, anti-inflammatory, and sedative effects of the CBD [19]. Β-caryophyllene interacts with the cannabinoid receptors and it is responsible for Cannabis anti-inflammatory properties [20]. Its anti-oxidant, anxiolytic, analgesic, and neuro-protective effects have been reported [21].

The concentration of these volatile molecules is affected by the extraction conditions at a high temperature. Moreover, the oxidation caused by UV light or heat during storage or transformation can determine the formation of oxygen-containing products as terpenoids and ketones [22,23]. In fact, secondary photo-oxidation of the terpenes can determine the generation of unstable allylic hydroperoxides whose reduction and subsequent oxidation can lead to the formation of alcohols and then aldehydes or ketones [24]. These compounds are studied for their potential toxicity, inherent to the promotion of oxidative stress [25,26].

A standardized sample preparation is therefore fundamental for extracting cannabinoids, preserving volatile terpenes and obtaining the right balance between the required decarboxylation (i.e., preheating Cannabis flos increases the concentration of active cannabinoids) and undesirable degradation in the volatile profile [27]. An optimized and homogenous final product is needed to guarantee the therapeutic continuity of the patients and also to reduce safety concerns related to over- or under-cannabis dose. In fact, medical cannabis is well tolerated but the risk of reported adverse effects (e.g., confusion, dizziness, drowsiness, hallucinations, euphoria, nausea, vomiting, and diarrhea) can increase in the fast upscale of the dosage and in the sub-set population with altered pharmacokinetics such as the elderly, as well as for the use of inappropriate preparation of medical Cannabis [28].

For oral-administered Cannabis, the extracts in different oil media have shown a great ease to modulate the dose and a good bioavailability of the product [11]. In particular, medium-chain triglycerides (MCT) oil is actually considered more suitable for extracting a stable product with an increased concentration of bioactive compounds than the olive oil, often employed as solvent [29]. Several extraction methods have been set up, including a decarboxylation step to boost the transformation of Δ9-THCA in Δ9-THC [30,31,32]. Moreover, different temperatures and time have been applied aiming to avoid terpene evaporation and/or degradation. 

In this context, we propose a new standardized and optimized method to obtain a MCT oil extract of Cannabis flos with a high content of bioactive compounds as Δ9-THC and terpenes and the lowest rate of degraded/oxidized products. The protocol was compared with other two reported procedures by the means of HPLC-ESI-MS/MS (cannabinoid analysis) and both HS-SPME-GC-MS and GC-FID (terpene analysis) techniques. This effort can overcome the challenge of having an advisable method to produce a high-value medical Cannabis oil.

## 2. Materials and Methods

### 2.1. Chemicals and Solvents

Medium-chain triglyceride (MCT) oil (Labrafac Lipophile WL 1349; Lotto: W2105378) was used as the extraction solvent. Analytical standards (Table S1) were purchased from Sigma–Aldrich S.p.A. (Milan, Italy): THC Cannabinoids Mixture-3 solution (multi-component certified solution standard of (-)-Δ9-THC (CAS 1972-08-3), cannabinol (CBN, CAS 521-35-7) and cannabidiol (CBD, CAS 13956-29-1) 1.0 mg/mL in methanol), THCA (CAS 23978-85-0) and CBDA (CAS 1244-58-2) 1 mg/mL in acetonitrile, internal standards (IS) Proadifen-SKF 525A (CAS 62-68-0) and *tert*-Butanol (CAS 75-65-0), Cannabis Terpene Mix-A (CRM40755) and Mix-B (CRM40937), β-Caryophyllene (CAS 87-44-5), Limonene (CAS 138-86-3) and α-Pinene (CAS 80-56-8) solutions (2.0 mg/mL in methanol). Solvents were supplied by VWR™ International, LLC, including methanol, 2-propanol, acetonitrile, and formic acid.

### 2.2. Plant Material and Extraction of Cannabis Oils

Moreover, 1 batch (n 20i07EY20j07) of medical Cannabis flos Bedrocan^®^ (THC 22% CBD < 1%) from Bedrocan International (Veendam, The Netherlands) was used for this study. Plant material was stored at room temperature or −20 °C until used. A total of 2 different extraction procedures were performed according to previous published studies [30,32] to obtain medical Cannabis oil 1 (MCO-1) and MCO-2 (SIFAP and Calvi methods, respectively). Furthermore, 1 new optimized method (CERFIT method) was specifically applied for the present study to obtain MCO-3. Briefly, 5 g of Cannabis flos were decarboxylated in a fully automated microwave extractor (Ethos X—advanced Microwave Extraction System, Milestone, FKV SRL, Italy; https://www.milestonesrl.com/industries/cannabis-and-terpenes (accessed on 20 January 2022)) set at 1500 Watt to reach and maintain a T = 100 °C. Cyclic decarboxylation (total time 8 h) was “heat-and-cool” performed in the following mode for five times (Table 2).

A hermetically closed flask was kept at −25 °C for 60 min, and then the extraction solvent was added to Cannabis flos: MCT oil (50 mL) was used for all preparations to minimise matrix effects. Extraction was carried out by turbo emulsion of the solution (Cannabis flos in MCT oil) for 5 min and sonication (200 W/30 kHz) with probe inserted in the oil flask for 8 min. During sonication time, the system was kept in a refrigerated water bath and the temperature was controlled with one thermometer in the water bath and one in the flask. For each method, 10 samples were prepared starting from 5 g of cannabis flos in 50 mL of MCT oil. All final oil samples (50 mL) were protected from the light in amber glass bottles and then stored at 4 °C.

### 2.3. Sample Preparation for Cannabinoid Analysis

MCO samples for High Performance Liquid Chromatography (HPLC) tandem Mass Spectrometry (MS/MS) analysis were prepared by dissolving 10 mg of Cannabis oil in 1.0 mL of 2-propanol. A measurement of 50 µL of each sample was then added to 950 µL of acetonitrile and 100 ng of IS; 2 µL were injected. An analysis was performed in duplicate.

### 2.4. HPLC-MS/MS Analysis of Cannabinoids

Analyses were assessed using a HPLC–MS-MS TSQ Fortis II (Thermo Fisher Scientific, San Jose, CA) equipped with a Surveyor MS quaternary pump with degasser, Surveyor AS auto-sampler and oven with Rheodyne valve with a 20-µL loop. For chromatographic separation, we used a HPLC column with reversed phase Agilent Poroshell 120 C-18 2.7 µm–4.6 × 150 mm. The mobile phase utilized for the gradients (Table 3) was composed of water at 0.1% formic acid (Solvent A) and methanol (Solvent B). Instrumental conditions for TSQ Fortis II are summarized in Table 4. The multiple reaction monitoring (MRM) acquisition mode was applied and the selected transitions are reported in Table 5.

### 2.5. Sample Preparation for Terpene Analysis

The extraction of total terpenes from Cannabis oils was carried out by headspace-solid phase microextraction (HS-SPME). A CAR/PDMS/DVB fibre was employed. Before the analysis, 500 mg of each oil sample were placed into a headspace vial along with 200 µL of the IS (*tert*-Butanol 0.02%) and incubated at 80 °C for 1 h. Then, 500 µL of gas were analysed by gas chromatography (GC) with a mass spectrometer (MS). After each analysis, the fibre was reconditioned for 5 min at 250 °C to prevent any contamination [30].

### 2.6. GC/MS Analysis of Terpenes

Analyses were performed with a Trace GC Ultra (Thermo-Fisher Scientific, Waltham, MA, USA) equipped with an STABILWAX-MS column (30 m × 0.25 mm i.d. 0.25 µm film thickness; Restek, Bellefonte, PA, USA), a quadrupole mass spectrometer Trace DSQII (ThermoFisher Scientific, Waltham, MA, USA). The injector temperature, in selected split mode 10:1, was 250 °C and those of the detector and transfer line were 250 and 210 °C, respectively. The oven temperature program was: 35°C 5 min, from 35 to 60 °C at 4 °C/min, then from 60 to 160 °C at 6 °C/min, from 160 to 200 °C at 20 °C/min and finally 200 °C at 20 min [32]. The quantification of identified terpenes was performed with a calibration curve (y = bx + a) of each relative standard (i.e., β-Caryophyllene, Limonene and α-Pinene). Calibrations curves were reported in Figures S1–S3. MS spectra of eluting peaks were compared with those of terpene standard mixtures to characterize and divide the terpenes according to their isoprene structure. Quantification of total mono/di/triterpenes, total sesquiterpenes and total oxidized terpenes was calculated summarizing a single quantity of each terpene of the same chemical class.

### 2.7. Statistical Analysis

Concentrations of cannabinoids and terpenes in analysed MCOs were expressed both as mean value and related standard deviation (SD), and as median value and range (min–max). Differences in mean and median concentrations among different MCOs were tested using the One-way ANOVA followed by Tukey–Kramer post-hoc analysis and the Kruskal-Wallis test followed by Dunn’s multiple comparisons test, respectively. Statistical analysis was performed using the Software STATA version 17. Statistical significance was considered for *p*-value < 0.05.

## 3. Results

### 3.1. Extraction Methods for Medical Cannabis Oils

In this work, two methods previously described in the literature for MCO preparation were applied [30,32]. They included a decarboxylation step at 115 °C for 40 min and 130 °C for 30 min, respectively. In the first method, the plant material was extracted by maceration at 100 °C for 40 min [32] whereas in the second method, the extraction was carried out for 30 min by sonication at controlled temperature [30]. MCT oil was shown to extract and preserve a significant concentration of terpenes compared to olive oil [29]. For this reason, we chose to use MCT oil for all methods in order to minimise differences due to matrix effects. In addition to these methods, one novel extraction procedure (CERFIT method) was developed in this work. The plant material was stored at −20 °C until use; therefore, it was put in a fan oven at 25 °C for 60 min before weighing. The decarboxylation of the plant material was carried out at a lower temperature (100 °C) for a shorter time (10 min) in CERFIT than in the other two methods: this step was repeated five times in order to alternate heating and cooling to preserve the phytocomplex. Moreover, the closed flask was kept under controlled vacuum to avoid the loss of volatile compounds. At the end of the cyclic process, the flask was maintained at −25 °C for 60 min recovering essential oil volatile components. Then, the extraction was performed by placing a probe sonicator in the flask (MCT oil plus Cannabis flos) and the system in a refrigerated water bath. Finally, the product was filtered and packaged. 

Ten cannabis samples were extracted with each method and the experimental conditions are compared in Table 6. Starting from 5 g of cannabis flos in 50 mL of MCT oil, a final product of 50 mL of medical cannabis oil was obtained. Cannabinoid and terpene profile was analysed for all of the samples (10 samples for each method).

### 3.2. Cannabinoids in Medical Cannabis Oils

**Figure 1:**
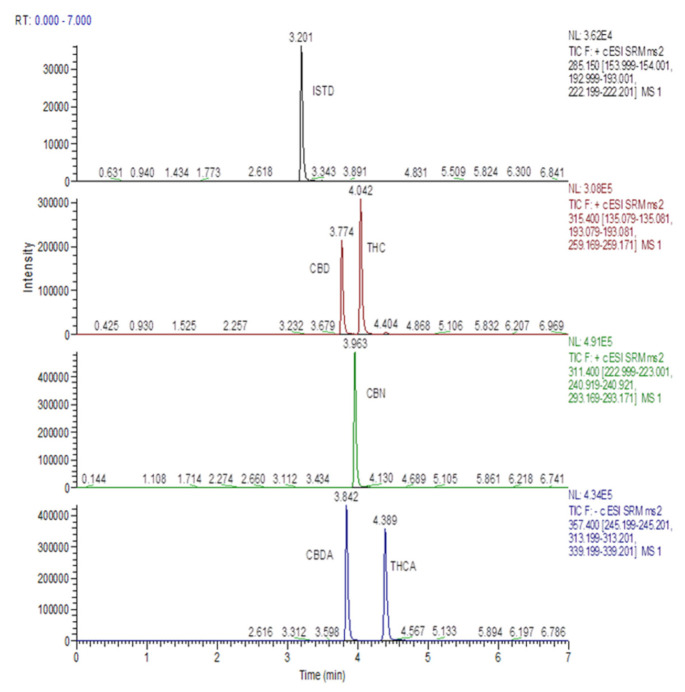
HPLC-MS/MS mass chromatogram of a standard calibration point for analytes involved in the study.

Concentrations of CBD, CBDA, CBN, Δ9-THC, and Δ9-THCA in MCO preparations are reported in Table 7. A representative HPLC-ESI-MS/MS chromatogram of a standard calibration point for the analytes is shown in Figure 1. Cannabinoids amounts were found in line with data available in the literature for Bedrocan^®^ flos preparations [30,31,32].

**Figure 2:**
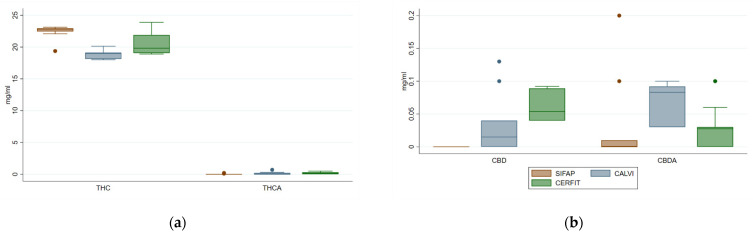
Distribution of concentrations of Δ9-THC, Δ9-THCA (panel (**a**)), CBD and CBDA (panel (**b**)) in medical Cannabis oil preparations extracted by three different methods.

Neutral psychoactive Δ9-THC concentration was high and consistent through all Cannabis oils (18.0–23.9 mg/mL), conversely its acidic precursor Δ9-THCA was under-represented (0.0–0.7 mg/mL) depending on the presence of a decarboxylation step (Figure 2a). The comparison of the 3 MCOs showed that MCO-1 had 9% of Δ9-THC more and 18% less of Δ9-THCA than MCO-2 and MCO-3.

SIFAP method failed to extract CBD and its acid precursor CBDA that were slightly recovered with the two other procedures (0–0.13 and 0–0.20 mg/mL, respectively). CBN, analysed to monitor eventual degradation/oxidation of Cannabis oils [33], was present in low amount (<0.20 mg/mL) in MCO1 and 2, while MCO3 showed a higher range (0.02–1.06 mg/mL) than the others (Figure 2b).

Overall, the three methods performed similarly in terms of final quantitative yield of extracted cannabinoids.

### 3.3. Terpenes in Medical Cannabis Oils

**Figure 3:**
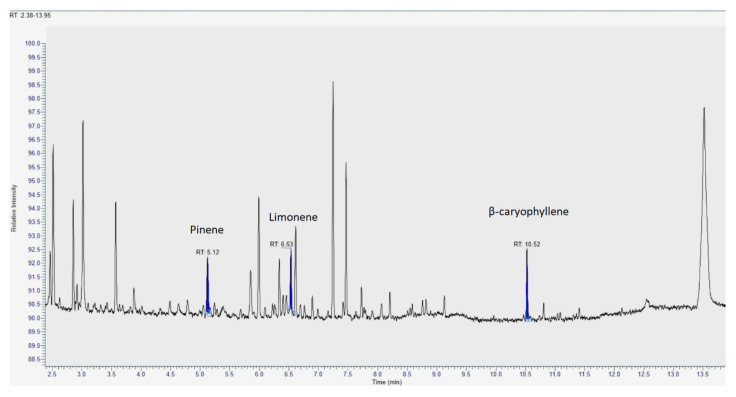
Representative GC/MS chromatograms of the extracts obtained from medical Cannabis.

The analysis of volatile compounds in the three MCOs was performed by means of HS-SPME coupled with both GC-FID and GC-MS (Figure 3). Identified volatiles were found in line with data available in the literature for Bedrocan^®^ flos preparations [11,30]. The quantitative data of the MCOs is shown in Table 8.

**Figure 4:**
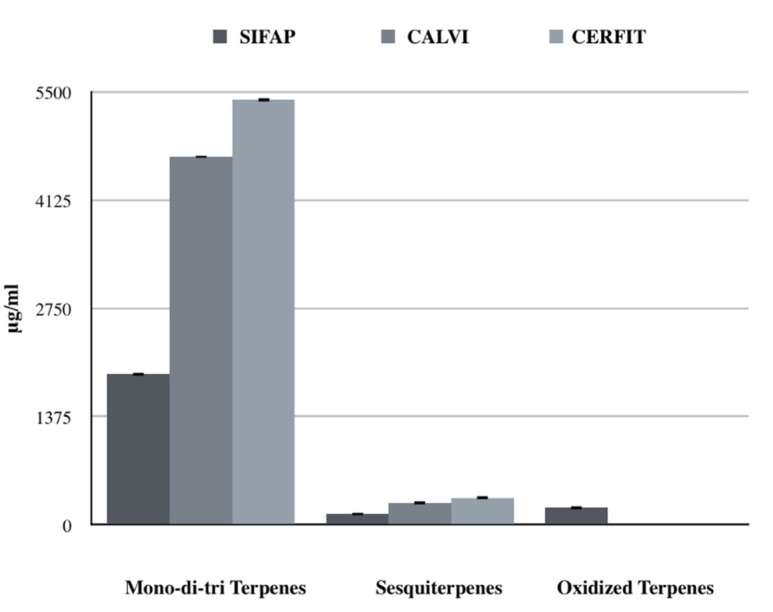
Comparison of terpene classes’ concentrations (μg/mL) obtained with the three different extraction method.

**Figure 5:**
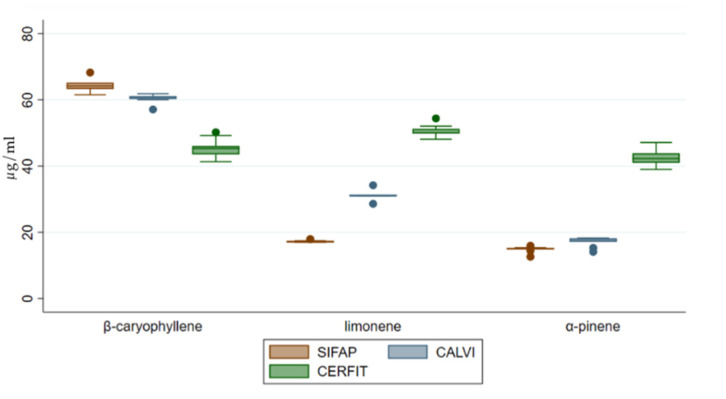
Distribution of concentrations of β-caryophyllene, limonene and α-pinene in medical Cannabis oil preparations extracted by three different methods.

Mono-di-tri terpenes were highly represented in all of the samples, accounting for 84–94% of total identified terpene concentration of each MCO with an increase by two or three times for MCO-2 and MCO-3, respectively compared to MCO-1 (Figure 4). Quantified molecules belonging to this chemical class were α-pinene and limonene showing a significant increasing trend from MCO-1 to MCO-3 (*p* value < 0.001; Figure 5). 

Sesquiterpenes were about 6% of the total identified terpenes in each MCO; MCO-3 had a significantly increased concentration compared to MCO-2 and MCO-1 (*p* value < 0.001; Figure 4). Interestingly, MCO-3 and MCO-2 were characterized by a lower presence of β-caryophyllene (13 and 22%, respectively) than MCO-1 (48%). These data could indicate a different qualitative volatile profile for the samples MCO-3, obtained with the CERFIT method (Figure 5). 

Oxidized terpenes, usually produced by photo-oxidation of primary terpenes [30], were only detected in MCO-1 (9.6% of the total terpenes; Figure 4).

Overall, the three methods showed a similar performance in terms of final quantitative yield of extracted cannabinoids. On the other hand, samples extracted with CERFIT methods displayed a distinctive profile highly rich in terpenes and devoid of oxidized volatile products.

## 4. Discussion

Here, we compared the concentrations of cannabinoids and terpenes of three MCOs obtained with three different extraction procedures in order to improve the therapeutic profile of the final product. In particular, quantitative analyses of terpenes were performed while several studies on MCOs did not investigate cannabis volatile compounds or performed qualitative or semi-quantitative approaches [31,32]. 

For the development of the extraction protocol, this work mainly focused on temperatures and times of the decarboxylation and following steps. It is well known that the decarboxylation (pre-heating) of the starting plant material is necessary for the conversion from acid to neutral cannabinoid forms [34]. In fact, cannabinoid concentrations in the 3 MCOs (all methods including pre-heating of at least 100 °C) were found to be consistent with previous findings and in line with the quantities declared by the manufacturer (Bedrocan) in the plant material [30,31,32]. On the other hand, the application of high temperatures for a long time increased the risk of thermal volatile evaporation/degradation [35]. Thus, our working hypothesis was that an improved extraction method had to include a decarboxylation step resulting in high cannabinoid concentration and minimal loss of terpenes. The innovative CERFIT method decarboxylated Cannabis flos at a moderate temperature (max 100 °C) in a microwave-assisted vacuum (to preserve the preparation from the external humidity) system: microwave was reported more effective than conventional extraction [35]. Moreover, we introduced a cyclical variation of the temperature in this preheating step in order to have rotation between heating (max 100 °C for 10 min) and cooling (100–25 °C for 25 min) and rest (25 °C for 55 min) reducing the thermal stress of the phyto-complex. In addition to SIFAP and CALVI procedures, a final step at −25 °C for 60 min was added to condense the evaporated components with no loss of volatile compounds. Moreover, it is important to highlight that our method permitted the full recovery of final MCO (i.e., starting from 5 g of Cannabis flos in 50 mL, we obtained 50 mL medical cannabis oil): this yield (100%) is given by a constant control of the filtration system applied before packaging. Finally, we could make a case for the good scalability of CERFIT method. In fact, we developed the present work starting from 5 g of Cannabis in 50 mL but we also conducted several experiments (data not shown) with major quantities (up to 30 g of Cannabis in 300 mL) maintaining the same Cannabis/matrix ratio (1:10) and the results overlapped both in terms of recovery and quali-quantitative cannabinoid and terpene profiles.

A significant increasing trend resulted in the total terpene concentrations of the MCOs extracted with the three methods (SIFAP < CALVI < CERFIT). This finding could be also determined by the utilized extraction technique: ultrasound-assisted extraction was reported more effective than conventional procedures [36]. Moreover, in the CALVI method, ultrasounds were applied for 30 min at the water bath containing the flask with inflorescences in MCT oil. CERFIT method used a probe sonicator inside the flask maintained in a refrigerated water bath. At the end of the extraction the water baths had a T of about 45 °C and 21 °C, respectively, and this difference could explain the major efficacy of CERFIT method since lower temperatures were reported to enhance the cavitation and consequently the yield of the extracted bioactive compounds [37].

Interestingly, total sesquiterpenes increased in MCO extracted with CERFIT method while β-caryophyllene concentration decreased. Thus, the extraction method appeared to influence the qualitative composition of the final oil product as well as the quantitative profile of volatile compounds.

Remarkably, the final composition of MCO-2 and MCO-3 was further varied. In particular, they were deprived of oxidized terpenes, thus free of secondary thermal oxidation’s products as aldehydes and ketones [30,38]. The formation of these compounds could significantly affect the quality of a product being a marker of aging [38]. For example, the presence of oxidized terpenes in essential oils has been associated with shifting colors, unpleasant smell, unpalatable taste, and consistency alterations [39]. Terpene oxidation may produce skin sensitizers causing hazardous allergic reactions in sensitive individuals [40,41,42]. Generally, the toxicity of terpenes and their oxidized derivatives is attributed to their pro-oxidant power originating reactive oxygen species (ROS) formation via lipid peroxidation [43]. For example, limonene acute toxicity is reported associated with its oxidized product that is limonene hydroperoxide [44]. Thus, the absence of oxidized components in MCOs is an important requisite both for the quality control of the product and the safety of the patients [38].

Finally, evidence results on the synergic role of Cannabis terpenes and cannabinoids to date were conflicting [45], but no studies investigated the effects of medical Cannabis preparations rich in hydrocarbon terpenes with no oxidant compounds, potentially harmful constituents [46,47]. 

In our opinion, the new extract is very promising for both the biological activity of the individual terpenes and the related entourage effect [48]. In fact, the most important Cannabis terpenes (limonene, pinene, myrcene, β-caryophyllene and linalool) were reported to be individually responsible for the anti-inflammatory, analgesic, antinociceptive, anxiolytic, and multi-target effects [49]. Concurrently, the synergistic effect between the phytocannabinoids [49,50] and terpenes and phytocannabinoids [51] was shown. In this context, a differential cannabinoid receptor activity [52] and the inhibition of p-glycoprotein [52,53] by terpenes were investigated. Moreover, several studies showed terpene enhancer activity on the absorption of active substances [54,55], as in the case of analgesics and anti-inflammatories [56,57]. For example, limonene and bisabolol were studied as enhancers in the percutaneous absorption of sumatriptan [58] and naproxen skin absorption was increased by linalool [59]. Furthermore, a cannabis preparation containing the most important terpenes could exert a multi-target synergy [60]. In fact, each component of the phyto-complex could have low therapeutic potency but their simultaneous pharmacological action was often shown to be highly effective with significantly low toxicity [61]. Thus, it is important to obtain concentrated cannabis oil preparations and maintain their terpene content. 

## 5. Conclusions

CERFIT extraction protocol was able to obtain a MCO composition increased in total mono-di-tri terpenes and sesquiterpene concentrations and with no oxidized terpenes. This rich bioactive compound product has the full potential to show important therapeutic improvements in essential future clinical investigations.

## Figures and Tables

**Table 1:** Main classes of compounds of *Cannabis sativa* (2D structure images are available at https://pubchem.ncbi.nlm.nih.gov/#query=CID%20number (accessed on 20 January 2022).

Compound Class	2D Structure Image	PubChem Identifier: CID	Medicinal Properties	Reference
Cannabinoids	C₂₁H₃₀O₂			
*Δ9-THC*	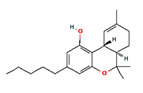	16078	*Psychoactive* Antinociceptive, antiemetic, appetite stimulator, anti-inflammatory	[9]
*CBD*	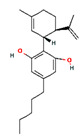	644019	*Nonpsychoactive* Anticonvulsant, analgesic, anti-inflammatory, antiemetic, anxiolytic, neuroprotective, sleep-promoting	[10]
**Flavonoids**	**C_6_C_3_C6**			
*apigenin*	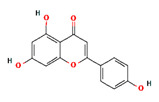	5280443	Anti-inflammatory, anxiolytic	[13]
*cannflavin B*	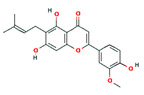	403815	Anti-inflammatory	[14]
**Terpenes**				
Monoterpenes	**C_10_H_16_**			
*α-pinene*	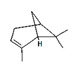	6654	Neuroprotective	[18]
*limonene*	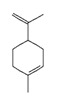	22311	Anti-inflammatory, anxiolytic, sedative	[19]
*myrcene*	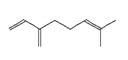	31253	Anti-inflammatory, analgesic, anxiolytic	[13]
Diterpene	**C_20_H_32_**			
*phytol*	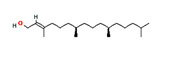	5280435	Sleep-promoting	[13]
Triterpenes	**C_30_H_48_**			
*friedelin*	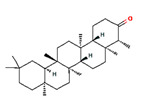	91472	Anti-inflammatory, analgesic	[17]
**Sesquiterpenes**	**C_15_H_24_**			
*α-humulene*	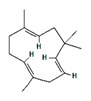	5281520	Anti-inflammatory, analgesic	[13]
*β-caryophyllene*	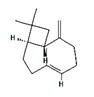	5281515	Anti-inflammatory, antioxidant, anxiolytic, analgesic, neuro-protective	[20,21]

**Table 2:** Cyclic decarboxylation applied in the CERFIT method.

Phase	Temperature °C (Microwave Power)	Time (min)	Cycles
Pre-heating	from 25 to 100 (1500 Watt)	5	5
Decarboxylation	100 (1500 Watt)	10	
Cooling	from 100 to 25	25	
Rest	25	55	

**Table 3:** Solvent Gradients for HPLC–MS-MS Analysis.

Time (min)	Solvent A (%)	Solvent B (%)
0.00	90	10
0.10	90	10
2.00	10	90
5.00	10	90
5.10	90	10
7.00	90	10

**Table 4:** Instrumental Conditions for HPLC–MS-MS Analysis.

Electrospray Ionization Source	ESI
Ion Transfer Tube temperature	350 °C
Vaporization temperature	300 °C
Electrospray tension	3.50 kV
Scanning acquisition	SIM
Isolation range	2 *m*/*z*

**Table 5:** MRM transitions of target cannabinoids.

Compound	Retention Time (tR, min)	MRM Transitions (*m*/*z*)		Collision Energy (eV)	Ion Spray Voltage (V)	Dwell Time (ms)
CBDA	3.84	357.4	245.2	28	3500	19.96
			313.2	22		
			339.2	19		
Δ9-THCA	4.39	357.4	245.2	28	3500	19.96
			313.2	22		
			339.2	19		
CBD	3.77	315.4	135.1	20	3500	19.96
			193.1	22		
			259.2	19		
Δ9-THC	4.04	315.4	135.1	20	3500	19.96
			193.1	22		
			259.2	19		
CBN	3.96	311.4	223.00	20	3500	19.96
			240.92	18		
			293.17	17		
ISTD	3.2	285.1	154.0 193.0 222.2			

**Table 6:** Detailed comparison of the three methods used for Cannabis oil preparation in this study.

METHOD	SIFAP [32]	CALVI [30]	CERFIT Present Work
Cannabis variety (5 g)/(T)	Bedrocan (RT)	Bedrocan (RT)	Bedrocan (−20 °C)
Matrix (50 mL)	MCT oil ^a^	MCT oil ^a^	MCT oil
Cannabis/Matrix ratio	1:10	1:10	1:10
Thawing	No	No	Yes/25 °C, 60 min fan oven
Decarboxylation step	Yes/115 °C, 40 min static oven	Yes/135 °C, 30 min fan oven	Yes/cyclic T ^b^, 8 h vacuum microwave extractor
Freezing	No	No	Yes/−20 °C, 60 min
Extraction process	Turbo emulsion water bath (100 °C 40 min)	Turbo emulsion bath sonicator (35 KHz 30 min)	Turbo emulsion probe sonicator (200 W/30 KHz 8 min)
Oil heating step	Yes	No ^c^	No ^d^
Filtration	Yes/filter paper	Yes/filter paper	Yes/filter paper
Preparation time (hour)	2	1.5	9.5 (8 nightlong)

^a^ modified from the original work where the used solvent was olive oil Ph. ^b^ cycles as described in Material and methods. ^c^ final temperature of the water in bath sonicator was 45 °C. ^d^ final temperature of the oil flask was 15 °C. T: temperature; CT: cyclic temperature; RT: room temperature.

**Table 7:** Cannabinoids (mg/mL) in MC extracts. Data from ten separate experiments each performed in triplicate. Tukey-Kramer (for One-Way ANOVA) or Dunn (for Kruskal-Wallis test) post-hoc analyses were applied. Differences were considered significant when *p* < 0.05 and are indicated with different superscripts letters: in each line, values with a different letter (a, b or c for mean values; d, e or f for median values) are significantly different (*p* < 0.05).

Compound	Overall		MCO-1 SIFAP		MCO-2 CALVI		MCO-3 CERFIT			
mg/mL	Mean (SD)	Median (Min–Max)	Mean (SD)	Median (Min–Max)	Mean (SD)	Median (Min–Max)	Mean (SD)	Median (Min–Max)	F Test *p* Value *	Chi2 Test *p* Value *°*
**CBDA**	0.04 (0.05)	0.03 (0.00–0.20)	0.03 (0.67)	0.00 ^d^ (0.00–0.20)	0.07 (0.03)	0.08 ^e^ (0.03–0.10)	0.03 (0.03)	0.03 ^d^ (0.00–0.10)	2.70 0.0854	8.13 0.0172
**CBD**	0.03 (0.04)	0.02 (0.00–0.13)	NQ	NQ	0.03 (0.05)	0.02 (0.00–0.13)	0.06 (0.02)	0.05 (0.04–0.09)	2.92 0.1044	3.86 0.0500
**CBN**	0.29 (0.35)	0.17 (0.02–1.06)	0.18 ^a^ (0.01)	0.18 (0.16–0.19)	0.17 ^a^ (0.14)	0.14 (0.04–0.54)	0.53 ^b^ (0.53)	0.55 (0.02–1.06)	4.33 0.0234	1.62 0.4447
**Δ9-THCA**	0.11 (0.18)	0.00 (0.00–0.70)	0.03 (0.67)	0.00 ^d^ (0.00–0.20)	0.14 (0.23)	0.01 ^d^ (0.00–0.70)	0.17 (0.18)	0.15 ^e^ (0.00–0.50)	1.84 0.1789	3.42 0.1809
**Δ9-THC**	20.58 (1.94)	19.77 (17.99–23.87)	22.42 ^a^ (1.11)	22.82 ^d^ (19.37–23.11)	18.80 ^b^ (0.72)	19.00 ^e^ (17.99–20.13)	20.52 ^c^ (1.75)	19.82 ^f^ (18.90–23.87)	20.32 0.0001	16.91 0.0002

NC not calculable. NQ: not quantifiable. * *p* value from One-way ANOVA ° *p* value from Kruskal–Wallis test.

**Table 8:** Terpenes in MCO extracts. Data from ten separate experiments each performed in triplicate. Tukey-Kramer (for One-Way ANOVA) or Dunn (for Kruskal-Wallis test) post-hoc analyses were applied. Differences were considered significant when *p* < 0.05 and are indicated with different superscripts letters: in each line, values with a different letter (a, b or c for mean values; d, e or f for median values) are significantly different (*p* < 0.05).

Compound	MCOs Overall		MCO-1 SIFAP		MCO-2 CALVI		MCO-3 CERFIT			
µg/mL	Mean (SD)	Median (Min–Max)	Mean (SD)	Median (Min–Max)	Mean (SD)	Median (Min–Max)	Mean (SD)	Median (Min–Max)	F Test *p* Value *	Chi2 Test *p* Value °
α-Pinene	24.82 (12.85)	17.23 (12.60–47.10)	14.86 ^a^ (0.88)	15.05 ^d^ (12.60–15.90)	17.10 ^b^ (1.38)	17.23 ^e^ (14.08–18.23)	42.50 ^c^ (2.41)	42.23 ^f^ (39.00–47.10)	834.73 0.0001	23.28 0.0001
Limonene	32.98 (13.96)	31.12 (16.99–54.39)	17.23 ^a^ (0.28)	17.17 ^d^ (16.99–17.94)	31.14 ^b^ (1.33)	31.12 ^e^ (28.58–34.20)	50.57 ^c^ (1.69)	50.05 ^f^ (48.11–54.39)	1786.70 0.0001	25.81 0.0001
β-Caryophyllene	56.77 (8.55)	60.87 (41.34–68.25)	64.36 ^a^ (1.73)	64.22 ^d^ (61.55–68.25)	60.53 ^b^ (1.31)	60.87 ^e^ (57.09–61.86)	45.41 ^c^ (2.74)	45.32 ^f^ (41.34–50.20)	245.76 0.0001	25.55 0.0001
Total mono- di-tri Terpenes	3998.60 (1528.39)	4678.89 (1914.10–5405.44)	1915.38 ^a^ (2.09)	1914.67 ^d^ (1914.09–1921.09)	4676.45 ^b^ (7.12)	4678.89 ^e^ (4656.38–4679.95)	5403.96 ^c^ (1.39)	5404.39 ^f^ (5401.80–5405.44)	1.8 × 10^6^ 0.0001	25.81 0.0001
Total Sesquiterpenes	248.18 (87.23)	274.70 (119.45–341.34)	132.6 ^a^ (4.82)	134.11 ^d^ (119.45–137.02)	274.27 ^b^ (1.63)	274.70 ^e^ (269.98–276.04)	337.65 ^c^ (1.97)	338.14 ^f^ (334.23–341.34)	1.1 × 10^4^ 0.0001	25.81 0.0001
Total oxidized TP	NC	NC	216.90 (2.60)	214.00 (214.00–223.00)	NQ	NQ	NQ	NQ	NC	NC
Total TP	4318.60 (1513.39)	4952.00 (2257.00–5743.00)	2264.50 ^a^ (4.40)	2263.50 ^d^ (2257.00–2273.00)	4950.10 ^b^ (7.20)	4952.00 ^e^ (4930.00–4954.00)	5741.20 ^c^ (1.32)	5741.00 ^f^ (5739.00–5743.00)	1.4 × 10^6^ 0.0001	25.81 0.0001

NC not calculable. NQ: not quantifiable. * *p* value from One-Way ANOVA ° *p* value from Kruskal–Wallis test.

## Data Availability

The data presented in this study are available in the article and in Supplementary Material and from the Corresponding Author upon reasonable request.

## Supplementary Materials

The following are available online at https://www.mdpi.com/article/10.3390/pharmaceutics14020298/s1, Figure S1: Calibration curve for quantification of β-caryophyllene, Figure S2: Calibration curve for quantification of limonene, Figure S3: Calibration curve for quantification of α-pinene, Table S1: Analytical standards used for the quantitative analyses of terpenes.

## References

[1] Crocq M.-A. (2020). History of Cannabis and the Endocannabinoid System. Dialogues Clin. Neurosci..

[2] Heng M., McTague M.F., Lucas R.C., Harris M.B., Vrahas M.S., Weaver M.J. (2018). Patient Perceptions of the Use of Medical Marijuana in the Treatment of Pain After Musculoskeletal Trauma: A Survey of Patients at 2 Trauma Centers in Massachusetts. J. Orthop. Trauma.

[3] Kosiba J.D., Maisto S.A., Ditre J.W. (2019). Patient-Reported Use of Medical Cannabis for Pain, Anxiety, and Depression Symptoms: Systematic Review and Meta-Analysis. Soc. Sci. Med..

[4] Aran A., Harel M., Cassuto H., Polyansky L., Schnapp A., Wattad N., Shmueli D., Golan D., Castellanos F.X. (2021). Cannabinoid Treatment for Autism: A Proof-of-Concept Randomized Trial. Mol. Autism.

[5] Uddin M.S., Mamun A.A., Sumsuzzman D.M., Ashraf G.M., Perveen A., Bungau S.G., Mousa S.A., El-Seedi H.R., Bin-Jumah M.N., Abdel-Daim M.M. (2020). Emerging Promise of Cannabinoids for the Management of Pain and Associated Neuropathological Alterations in Alzheimer’s Disease. Front. Pharmacol..

[6] García-Gutiérrez M.S., Navarrete F., Gasparyan A., Austrich-Olivares A., Sala F., Manzanares J. (2020). Cannabidiol: A Potential New Alternative for the Treatment of Anxiety, Depression, and Psychotic Disorders. Biomolecules.

[7] Stella B., Baratta F., Della Pepa C., Arpicco S., Gastaldi D., Dosio F. (2021). Cannabinoid Formulations and Delivery Systems: Current and Future Options to Treat Pain. Drugs.

[8] Filipiuc L.E., Ababei D.C., Alexa-Stratulat T., Pricope C.V., Bild V., Stefanescu R., Stanciu G.D., Tamba B.-I. (2021). Major Phytocannabinoids and Their Related Compounds: Should We Only Search for Drugs That Act on Cannabinoid Receptors?. Pharmaceutics.

[9] Pattnaik F., Nanda S., Mohanty S., Dalai A.K., Kumar V., Ponnusamy S.K., Naik S. (2022). Cannabis: Chemistry, Extraction and Therapeutic Applications. Chemosphere.

[10] Mlost J., Bryk M., Starowicz K. (2020). Cannabidiol for Pain Treatment: Focus on Pharmacology and Mechanism of Action. Int. J. Mol. Sci..

[11] Pavlovic R., Nenna G., Calvi L., Panseri S., Borgonovo G., Giupponi L., Cannazza G., Giorgi A. (2018). Quality Traits of “Cannabidiol Oils”: Cannabinoids Content, Terpene Fingerprint and Oxidation Stability of European Commercially Available Preparations. Molecules.

[12] Bettiol A., Lombardi N., Crescioli G., Maggini V., Gallo E., Mugelli A., Firenzuoli F., Baronti R., Vannacci A. (2018). Galenic Preparations of Therapeutic Differ in Cannabinoids Concentration: A Quantitative Analysis of Variability and Possible Clinical Implications. Front. Pharmacol..

[13] Baron E.P. (2018). Medicinal Properties of Cannabinoids, Terpenes, and Flavonoids in Cannabis, and Benefits in Migraine, Headache, and Pain: An Update on Current Evidence and Cannabis Science. Headache.

[14] Erridge S., Mangal N., Salazar O., Pacchetti B., Sodergren M.H. (2020). Cannflavins—From Plant to Patient: A Scoping Review. Fitoterapia.

[15] Sommano S.R., Chittasupho C., Ruksiriwanich W., Jantrawut P. (2020). The Cannabis Terpenes. Molecules.

[16] Bautista J.L., Yu S., Tian L. (2021). Flavonoids in: Biosynthesis, Bioactivities, and Biotechnology. ACS Omega.

[17] Antonisamy P., Duraipandiyan V., Ignacimuthu S. (2011). Anti-Inflammatory, Analgesic and Antipyretic Effects of Friedelin Isolated from Azima Tetracantha Lam. in Mouse and Rat Models. J. Pharm. Pharmacol..

[18] Miyazawa M., Yamafuji C. (2005). Inhibition of Acetylcholinesterase Activity by Bicyclic Monoterpenoids. J. Agric. Food Chem..

[19] Liktor-Busa E., Keresztes A., LaVigne J., Streicher J.M., Largent-Milnes T.M. (2021). Analgesic Potential of Terpenes Derived from. Pharmacol. Rev..

[20] Gertsch J., Leonti M., Raduner S., Racz I., Chen J.-Z., Xie X.-Q., Altmann K.-H., Karsak M., Zimmer A. (2008). Beta-Caryophyllene Is a Dietary Cannabinoid. Proc. Natl. Acad. Sci. USA.

[21] Hanuš L.O., Hod Y. (2020). Terpenes/Terpenoids in: Are They Important?. Med Cannabis Cannabinoids.

[22] Scurria A., Sciortino M., Presentato A., Lino C., Piacenza E., Albanese L., Zabini F., Meneguzzo F., Nuzzo D., Pagliaro M. (2020). Volatile Compounds of Lemon and Grapefruit IntegroPectin. Molecules.

[23] Booth J.K., Page J.E., Bohlmann J. (2017). Terpene Synthases from Cannabis Sativa. PLoS ONE.

[24] Marchini M., Charvoz C., Dujourdy L., Baldovini N., Filippi J.-J. (2014). Multidimensional Analysis of Cannabis Volatile Constituents: Identification of 5,5-Dimethyl-1-vinylbicyclo[2.1.1]hexane as a Volatile Marker of Hashish, the Resin of *Cannabis sativa* L. J. Chromatogr. A.

[25] Du Z.-C., Xia Z.-S., Zhang M.-Z., Wei Y.-T., Malhotra N., Saputra F., Audira G., Roldan M.J.M., Hsiao C.-D., Hao E.-W. (2021). Sub-Lethal Camphor Exposure Triggers Oxidative Stress, Cardiotoxicity, and Cardiac Physiology Alterations in Zebrafish Embryos. Cardiovasc. Toxicol..

[26] Chueca B., Pagán R., García-Gonzalo D. (2014). Oxygenated Monoterpenes Citral and Carvacrol Cause Oxidative Damage in Escherichia Coli without the Involvement of Tricarboxylic Acid Cycle and Fenton Reaction. Int. J. Food Microbiol..

[27] Booth J.K., Bohlmann J. (2019). Terpenes in Cannabis Sativa—From Plant Genome to Humans. Plant Sci..

[28] Crescioli G., Lombardi N., Bettiol A., Menniti-Ippolito F., Da Cas R., Parrilli M., Del Lungo M., Gallo E., Mugelli A., Maggini V. (2020). Adverse Events Following Cannabis for Medical Use in Tuscany: An Analysis of the Italian Phytovigilance Database. Br. J. Clin. Pharmacol..

[29] Ramella A., Roda G., Pavlovic R., Cas M.D., Casagni E., Mosconi G., Cecati F., Minghetti P., Grizzetti C. (2020). Impact of Lipid Sources on Quality Traits of Medical Cannabis-Based Oil Preparations. Molecules.

[30] Calvi L., Pentimalli D., Panseri S., Giupponi L., Gelmini F., Beretta G., Vitali D., Bruno M., Zilio E., Pavlovic R. (2018). Comprehensive Quality Evaluation of Medical *Cannabis sativa* L. Inflorescence and Macerated Oils Based on HS-SPME Coupled to GC-MS and LC-HRMS (q-Exactive Orbitrap^®^) Approach. J. Pharm. Biomed. Anal..

[31] Ternelli M., Brighenti V., Anceschi L., Poto M., Bertelli D., Licata M., Pellati F. (2020). Innovative Methods for the Preparation of Medical Cannabis Oils with a High Content of Both Cannabinoids and Terpenes. J. Pharm. Biomed. Anal..

[32] Pacifici R., Marchei E., Salvatore F., Guandalini L., Busardò F.P., Pichini S. (2017). Evaluation of Cannabinoids Concentration and Stability in Standardized Preparations of Cannabis Tea and Cannabis Oil by Ultra-High Performance Liquid Chromatography Tandem Mass Spectrometry. Clin. Chem. Lab. Med..

[33] Scholz C., Madry M.M., Kraemer T., Baumgartner M.R. (2021). LC-MS/MS Analysis of Δ9-THC, CBN and CBD in Hair: Investigation of Artefacts. J. Anal. Toxicol..

[34] Grotenhermen F. (2003). Pharmacokinetics and Pharmacodynamics of Cannabinoids. Clin. Pharmacokinet..

[35] Filly A., Fernandez X., Minuti M., Visinoni F., Cravotto G., Chemat F. (2014). Solvent-Free Microwave Extraction of Essential Oil from Aromatic Herbs: From Laboratory to Pilot and Industrial Scale. Food Chem..

[36] Mwaurah P.W., Kumar S., Kumar N., Attkan A.K., Panghal A., Singh V.K., Garg M.K. (2020). Novel Oil Extraction Technologies: Process Conditions, Quality Parameters, and Optimization. Compr. Rev. Food Sci. Food Saf..

[37] Wen C., Zhang J., Zhang H., Dzah C.S., Zandile M., Duan Y., Ma H., Luo X. (2018). Advances in Ultrasound Assisted Extraction of Bioactive Compounds from Cash Crops—A Review. Ultrason. Sonochem..

[38] Bitterling H., Lorenz P., Vetter W., Conrad J., Kammerer D.R., Stintzing F.C. (2020). Rapid Spectrophotometric Method for Assessing Hydroperoxide Formation from Terpenes in Essential Oils upon Oxidative Conditions. J. Agric. Food Chem..

[39] Turek C., Stintzing F.C. (2013). Stability of Essential Oils: A Review. Comp. Rev. Food Sci. Food Saf..

[40] Karlberg A.-T., Börje A., Duus Johansen J., Lidén C., Rastogi S., Roberts D., Uter W., White I.R. (2013). Activation of Non-Sensitizing or Low-Sensitizing Fragrance Substances into Potent Sensitizers—Prehaptens and Prohaptens. Contact Dermat..

[41] Karlberg A.-T., Lepoittevin J.-P. (2021). One Hundred Years of Allergic Contact Dermatitis due to Oxidized Terpenes: What We Can Learn from Old Research on Turpentine Allergy. Contact Dermat..

[42] Diepgen T.L., Ofenloch R., Bruze M., Cazzaniga S., Coenraads P.J., Elsner P., Goncalo M., Svensson Å., Naldi L. (2015). Prevalence of Fragrance Contact Allergy in the General Population of Five European Countries: A Cross-Sectional Study. Br. J. Dermatol..

[43] Agus H.H. (2021). Terpene Toxicity and Oxidative Stress. Toxicology.

[44] Chubukov V., Mingardon F., Schackwitz W., Baidoo E.E.K., Alonso-Gutierrez J., Hu Q., Lee T.S., Keasling J.D., Mukhopadhyay A. (2015). Acute Limonene Toxicity in Escherichia Coli Is Caused by Limonene Hydroperoxide and Alleviated by a Point Mutation in Alkyl Hydroperoxidase AhpC. Appl. Environ. Microbiol..

[45] Anand U., Pacchetti B., Anand P., Sodergren M.H. (2021). Cannabis-Based Medicines and Pain: A Review of Potential Synergistic and Entourage Effects. Pain Manag..

[46] Dosoky N.S., Setzer W.N. (2021). Maternal Reproductive Toxicity of Some Essential Oils and Their Constituents. Int. J. Mol. Sci..

[47] Perestrelo R., Silva C., Fernandes M.X., Câmara J.S. (2019). Prediction of Terpenoid Toxicity Based on a Quantitative Structure-Activity Relationship Model. Foods.

[48] Worth T. (2019). Cannabi’s Chemical Synergies. Nature.

[49] Weston-Green K., Clunas H., Jimenez Naranjo C. (2021). A Review of the Potential Use of Pinene and Linalool as Terpene-Based Medicines for Brain Health: Discovering Novel Therapeutics in the Flavours and Fragrances of Cannabis. Front. Psychiatry.

[50] Pamplona F.A., da Silva L.R., Coan A.C. (2018). Potential Clinical Benefits of CBD-Rich Extracts Over Purified CBD in Treatment-Resistant Epilepsy: Observational Data Meta-Analysis. Front. Neurol..

[51] Russo E.B. (2011). Taming THC: Potential Cannabis Synergy and Phytocannabinoid-Terpenoid Entourage Effects. Br. J. Pharmacol..

[52] Bilbrey J.A., Ortiz Y.T., Felix J.S., McMahon L.R., Wilkerson J.L. (2021). Evaluation of the Terpenes β-Caryophyllene, α-Terpineol, and γ-Terpinene in the Mouse Chronic Constriction Injury Model of Neuropathic Pain: Possible Cannabinoid Receptor Involvement. Psychopharmacology.

[53] Yoshida N., Takada T., Yamamura Y., Adachi I., Suzuki H., Kawakami J. (2008). Inhibitory Effects of Terpenoids on Multidrug Resistance-Associated Protein 2- and Breast Cancer Resistance Protein-Mediated Transport. Drug Metab. Dispos..

[54] Schmitt S., Schaefer U.F., Doebler L., Reichling J. (2009). Cooperative Interaction of Monoterpenes and Phenylpropanoids on the in Vitro Human Skin Permeation of Complex Composed Essential Oils. Planta Med..

[55] Yamane M.A., Williams A.C., Barry B.W. (1995). Terpene Penetration Enhancers in Propylene Glycol/water Co-Solvent Systems: Effectiveness and Mechanism of Action. J. Pharm. Pharmacol..

[56] Senyiğit T., Padula C., Ozer O., Santi P. (2009). Different Approaches for Improving Skin Accumulation of Topical Corticosteroids. Int. J. Pharm..

[57] Furuishi T., Kato Y., Fukami T., Suzuki T., Endo T., Nagase H., Ueda H., Tomono K. (2013). Effect of Terpenes on the Skin Permeation of Lomerizine Dihydrochloride. J. Pharm. Pharm. Sci..

[58] Femenía-Font A., Balaguer-Fernández C., Merino V., Rodilla V., López-Castellano A. (2005). Effect of Chemical Enhancers on the in Vitro Percutaneous Absorption of Sumatriptan Succinate. Eur. J. Pharm. Biopharm..

[59] Guo X., Rong Y., Zhang L., Ye J.-C. (2016). Enhancing Effect of Chiral Enhancer Linalool on Skin Permeation of Naproxen. Zhongguo Yi Xue Ke Xue Yuan Xue Bao.

[60] Sexton M., Shelton K., Haley P., West M. (2018). Evaluation of Cannabinoid and Terpenoid Content: Cannabis Flower Compared to Supercritical CO2 Concentrate. Planta Med..

[61] Pang M.-H., Kim Y., Jung K.W., Cho S., Lee D.H. (2012). A Series of Case Studies: Practical Methodology for Identifying Antinociceptive Multi-Target Drugs. Drug Discov. Today.

