# Supplementary material for: An Optimized Terpene Profile for a New Medical Cannabis Oil
Source: Pharmaceutics. 2022 Jan 27;14(2):298. doi: 10.3390/pharmaceutics14020298 (PMC8879232; doi:10.3390/pharmaceutics14020298)
Supplement: Supplementary file 1 [file pharmaceutics-14-00298-s001.zip › pharmaceutics-1561560-supplementary.pdf]

Supplementary Materials: An Optimized Terpene Profile for a New Medical Cannabis Oil

Valentina Maggini, Lorenzo Calvi, Tommaso Pelagatti, Eugenia Gallo, Celine Civati, Carlo Privitera, Flavio Squillante, Paolo Maniglia, Domenico Di Candia, Roberto Spampatti and Fabio Firenzuoli

Table S1. Analytical standards used in this work (SigmaAldrich)

Compound	CAS Number
**THC Cannabinoids Mixture-3 solution**	
CBD	13956-29-1
CBN	521-35-7
THC	1972-08-3
**Other Cannabinoids**	
CBDA	1244-58-2
THCA	23978-85-0)
**Monoterpenes**	
Limonene	138-86-3
alpha-pinene	80-56-8
**Sesquiterpenes**	
beta-caryophyllene	87-44-5
**Cannabis Terpene Mix A**	
beta-Pinene	127-91-3
Camphene	79-92-5
alpha-Pinene	80-56-8
3-Carene	13466-78-9
(R)-(+)-Limonene	5989-27-5
gamma-Terpinene	99-85-4
Citronellol	106-22-9
(+)-Pulegone	89-82-7
Geranyl acetate	105-87-3

Compound	CAS Number
alpha-Cedrene	469-61-4
Nerolidol	7212-44-4
**Cannabis Terpene Mix B**	
beta-Pinene	18172-67-3
beta-Caryophyllene	87-44-5
Phytol	
Limonene	138-86-3
Geraniol	106-24-1
(1S)-(+)-3-Carene	498-15-7
Linalool	78-70-6
p-Cymene	99-87-6
**Oxydized Terpene**	
4-Thujanol	546-79-2
1,8-Cineole	470-82-6
(±)-Camphor	76-22-2
(±)-Terpinen-4-ol	20126-76-5
α-Terpineol	98-554-5
Estragole	140-67-0
(1S)-(−)-Verbenone	1196-01-6

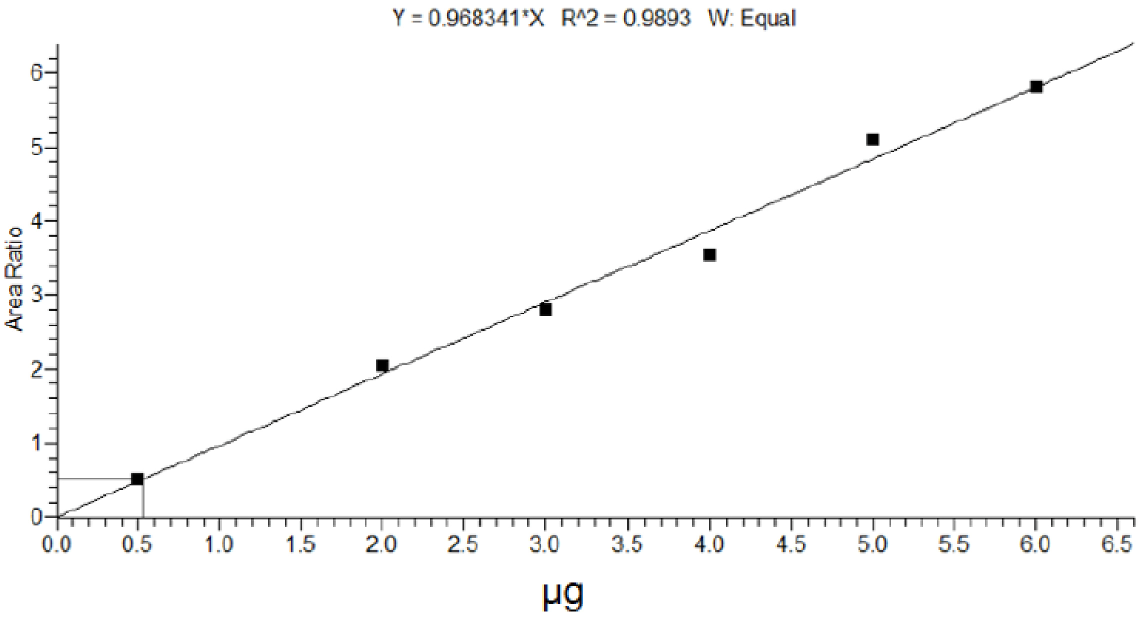

Figure S1. Calibration curve for quantification of β-caryophyllene

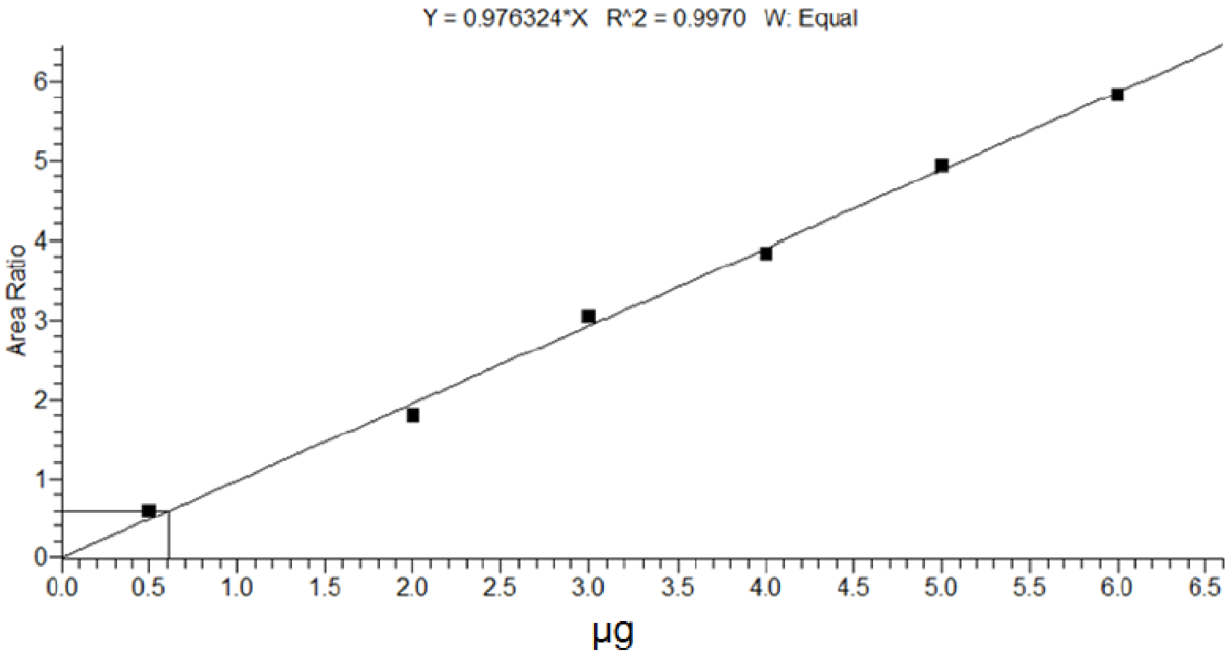

Figure S2. Calibration curve for quantification of limonene

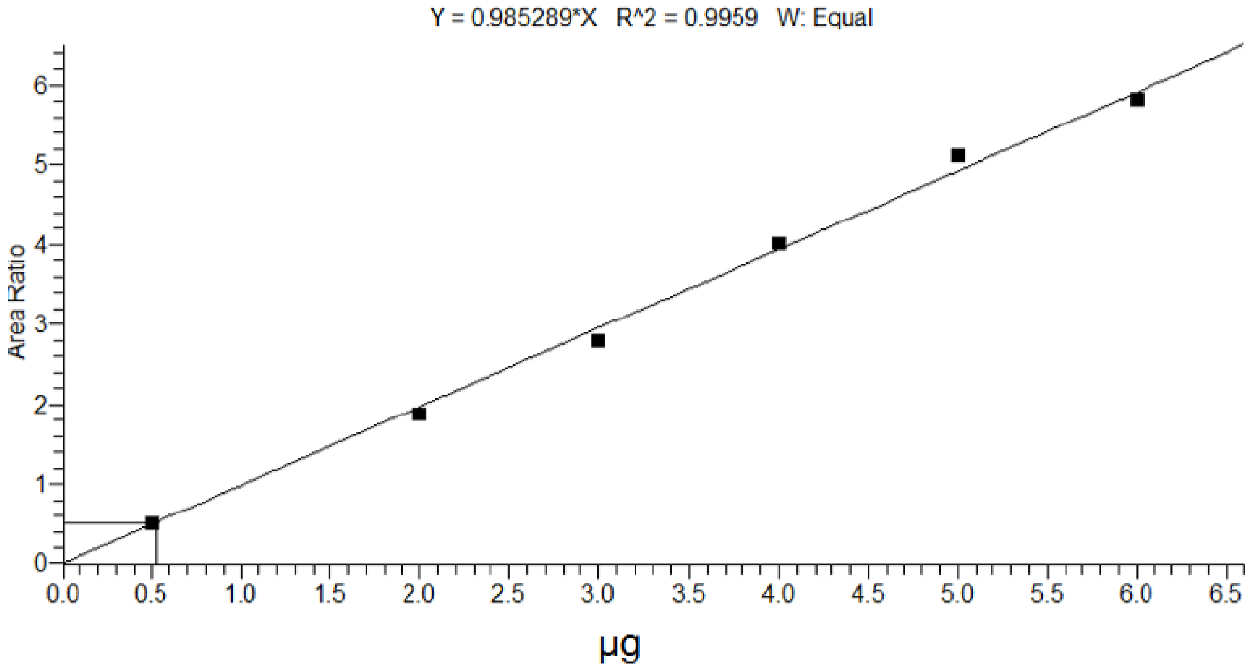

Figure S3. Calibration curve for quantification of α-pinene
